# Analysis and Authentication of Avocado Oil Using High Resolution NMR Spectroscopy

**DOI:** 10.3390/molecules26020310

**Published:** 2021-01-09

**Authors:** Fenfen Tang, Hilary S. Green, Selina C. Wang, Emmanuel Hatzakis

**Affiliations:** 1 Department of Food Science and Technology, The Ohio State University, Columbus, OH 43210, USA; tang.1263@buckeyemail.osu.edu; 2 Department of Food Science and Technology, University of California Davis, Davis, CA 95616, USA; hsgreen@ucdavis.edu (H.S.G.); scwang@ucdavis.edu (S.C.W.); 3 Olive Center, University of California Davis, Davis, CA 95616, USA; 4 Foods for Health Discovery Theme, The Ohio State University, Columbus, OH 43210, USA

**Keywords:** avocado oil, NMR, adulteration, purity

## Abstract

Avocado oil is a food product of high commercial and nutritional value. As a result, it can be a subject of adulteration similar to other high-value edible oils, such as olive oil. For olive oil and many other foods products, NMR spectroscopy has been successfully used for authentication and quality assessment. In this study, we apply NMR analysis to avocado oil to differentiate it from other oils including olive, canola, high-oleic (HO) safflower, HO sunflower and soybean oil using commercial and lab-made samples of avocado oils. NMR allowed the rapid analysis of the fatty acid profile and detection of minor compounds, such as sterols, oxidation products, and hydrolysis products, which can be used to assess oil quality and authenticity. The NMR assignment was conducted using traditional 2D NMR and the novel NOAH super-sequences. Combining chemometrics with NMR enabled us to differentiate between avocado oil and other oils. Avocado oil has compositional similarities with other vegetable oils, such as HO sunflower and HO safflower oil, which can be used as potential adulterants. Despite these similarities, NMR-based metabolomics captured differences in the levels of certain compounds including fatty acids, terpenes, sterols, and oxidation products to detect adulteration and for quality control purposes.

## 1. Introduction

Globally, consumers are placing more emphasis on health and delayed aging. They seek food products rich in nutrients and bioactive compounds associated with various health benefits. Avocado oil is a food product derived from the flesh of avocado fruit and has an increasing popularity among consumers because of the health-promoting effects associated with avocados. Similar to the fruit, avocado oil has a content of high oleic acid, a monounsaturated fatty acid, which may be responsible for the blood-pressure- and low-density lipoprotein (LDL)-reducing effects [1,2]. Mexico is the largest avocado-producing country, while the US is the largest avocado-consuming country [3]. Avocado oil is extracted using various methods, including cold-pressed, ultrasound-assisted aqueous extraction, supercritical CO_2_, and solvent extraction, among others [4]. Since it is a product of high commercial value with increasing consumer demand, avocado oil can be a subject of adulteration with cheaper oils. With questions arising about its safety and authenticity, rapid and efficient analytical methods that allow for fast and robust screening of a large number of samples in a high throughput manner are needed.

Although there are several studies focusing on increasing the extraction yield [5,6,7,8] and characterizing avocado oils based on region and cultivar [9,10,11,12], there are currently not many studies for the determination of its authenticity and quality control. Only recently, Green and Wang presented the first study for the determination of avocado oil purity [13]. In that paper, lipid components such as fatty acids (FA), sterols, and tocopherols were found to be biomarkers that can be used for avocado oil authentication, as found by using gas chromatography (GC)- and liquid chromatography (LC)-based analytical protocols. Another challenge with the analysis of avocado oil, and edible oils in general, is that most common analytical methods for the profiling of oils have their own limitations. For example, despite its advantages, GC is time-consuming, different extraction protocols are required for various classes of compounds, and lipid oxidation may take place during the analysis [14,15].

In response to these limitations, we introduce the application of high-resolution NMR spectroscopy combined with chemometrics for the untargeted analysis of avocado oil to achieve its characterization and verify authentication. NMR is a highly reproducible and non-destructive methodology that can be used for the rapid and efficient fingerprinting of avocado oil and its discrimination from other edible oils. NMR can be used independently or complementary to existing methodologies, such as GC, and therefore improve the arsenal of food quality control laboratories and regulatory agencies to guarantee authentication and establish a fair market system for consumers and producers. ^1^H-NMR has been used for the authentication and classification of foods including oils [16,17,18], but to our knowledge, this is the first application for avocado oil authentication. This research is expected to improve our capabilities for food evaluation and authentication purposes and to generate new methods for mixture analysis and food safety.

## 2. Results and Discussion

### 2.1. NMR Assignment of the Avocado Oil Spectra

Avocado oil is a mixture of various FA, such as oleic (OL), palmitoleic (PO), linoleic (LO), linolenic (LN), and saturated fatty acids (SFA), mainly in the form of triglycerides (TG). Minor compounds including sterols, oxidation products, and hydrolysis products, such as diglycerides (DG) and monoglycerides (MG), are also found. These compounds often have distinct diagnostic signals in the 1D-NMR spectrum, and their assignment is critical for the identification of biomarkers, driving clustering and classifications resulting from untargeted analysis. NMR allows the identification of these compounds in one snapshot without the need for any separation steps or any other type of sample preparation, which is an important asset compared to other analytical techniques.

**Figure 1:**
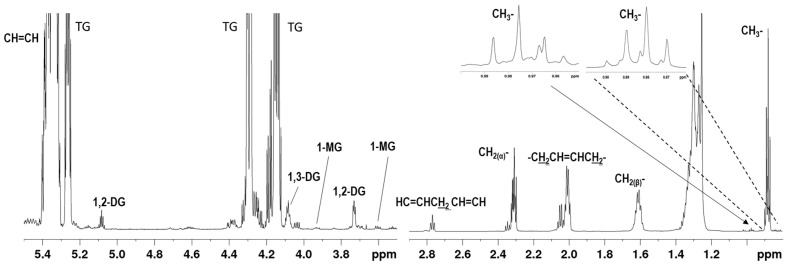
700 MHz ^1^H-NMR spectrum of avocado oil in CDCl_3_. The assignments of the labeled signals are presented in Table 1. The insets show the methyl proton signals of OL, LO, and LN. CH_2_α and CH_2_β, refer to the methylene protons alpha and beta to carboxyl group, respectively.

Figure 1 illustrates the ^1^H-NMR spectrum of an avocado oil sample with the resonances of the main acyl chains and glycerides. Table 1 shows the ^1^H-NMR chemical shifts of these constituents as identified in this study. The methyl groups of SFA, PO, and OL appear as a triplet at δ 0.879, partially overlapping with the triplet of LO at δ 0.883, while the methyl group of LN appears as a triplet at δ 0.976 because it is located closer to the double bond of the LN acyl chain. Distinct signals are also observed for the allylic protons of OL and LO at δ 2.00 and 2.05, respectively, while separate signals for the CH_2_α of triglycerides and diglycerides/free fatty acids with C4 were observed.

**Figure 2:**
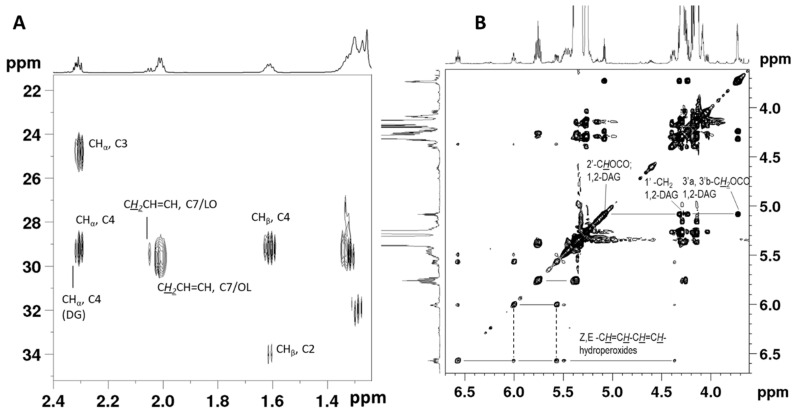
Signal assignment on an HMBC spectrum of avocado oil obtained using the NOAH-3 (BSC) super-sequence (**A**). Total Correlation Homonuclear Spectroscopy (TOCSY) spectrum of avocado oil in CDCl_3_ solution, showing connectivities between protons along common coupling pathways (**B**).

The chemical shifts were confirmed using traditional 2D NMR sequences and the novel NOAH (nested NMR by ordered acquisition using ^1^H detection) NMR super-sequences. NOAH experiments allow the generation of high-quality 2D-NMR data in a much faster manner [19]. This speed is achieved because they include one evolution period for all combined pulse sequences, resulting in a reduction of around 60% in the length of the experiment. NOAH experiments were recently used for pomegranate juice analysis [20], and to our knowledge this instance is the first application of the analysis for lipid mixtures. Figure 2A shows the HMBC spectrum obtained by NOAH-3 (BSC) super-sequence that combines three different 2D-NMR experiments, namely HMBC, HSQD-DEPT, and COSY. As shown, it is a spectrum of high quality, indicating the great potential of super-sequences for lipid analysis. The correlation peaks between CH_2_α and C3/C4 of acyl chains and the correlation peaks between CH_2_β and C2/C4 are shown.

Additional information about the NMR assignments, especially for minor compounds, was extracted by the Total Correlation Homonuclear Spectroscopy (TOCSY) spectrum shown in Figure 2B. TOCSY confirmed the presence of hydroperoxides with Z, E-conjugated double bonds formed by the reaction of molecular oxygen with unsaturated FA, and have signals at δ 6.570, 6.005, 5.569, and 5.496. Another signal, which appears as a multiplet at δ 5.755, has a cross peak in TOCSY with a signal at δ 5.380, a correlation peak in the NOAH HSQD-DEPT with a carbon at δ 136.74, and two correlation peaks at NOAH HMBC with ^13^C signals at δ 32.2 and 87.0 (Figure S1). This result indicates that the signal at δ 5.755 may belong to an olefinic proton located close to an oxygenated carbon; thus. it was also attributed to an oxidation product. The broad signals at δ 7.88 and 7.79 belong to OO*H* protons of hydroperoxides such as 16-OOH and 9-OOH; however, these chemical shifts are sensitive to hydrogen bonds and other sample conditions [21]; thus, comparisons with other samples and literature should be done with caution. TOCSY also shows the connections between the glyceridic protons of 1,2-DG, which share a common coupling pathway. H3’a/H3’b appear as a multiplet at δ 3.730, H1’a/H1’b resonates at δ 4.24 and 4.32, partially overlapping with triglycerides, and H’2 appears as a multiplet at δ 5.083. The signals at δ 3.73 and δ 4.08 for 1,2-DG and 1,3-DG, respectively, can be used for monitoring the isomerization of the 1,2-DG to the 1,3-DG and the determination of 1,2-DG/total DG, which are indicators of the thermal stress, freshness, and storage history of the oil [22,23].

The NMR signals of the sterolic fraction of avocado oil resonating from δ 0.3–0.7 as shown in Figure S2. The doublets at δ 0.339 (*J* = 4.20 Hz) and 0.576 (*J* = 3.75 Hz) belong to the CH_2_ of sterols bearing a cyclopropane ring, and it is attributed to the esters of cycloartenol. At δ 0.537, there is a singlet that belongs to campesterol, and the signal at δ 0.679 belongs to β-sitosterol. H3 of esterified sterols appears as multiplet of the hydrogen H3 of at δ 4.59–4.65 and has a ratio of 1:3 with the singlet at δ 1.02, and thus it can be attributed to C19 of esterified sterols.

### 2.2. Fatty Acid Profile

The FA profile and important factors such as the unsaturation degree (UD), which represents the total unsaturated FA and is equivalent to iodine value [24], can be calculated by NMR in a similar manner that has been described for other oils such as olive oil [25], coffee oil [26], and fish oil [27]. Nevertheless, one significant difference from other oils is that, for avocado oil, NMR is not able to distinguish between OL and PO, a 16:1 ω-7 acid that is found in relatively small amounts in avocado oil and has allylic protons that overlap with those of OL. The proportions of FA and the unsaturation degree in avocado oil can be determined using the following relationships:

$$C_{LN}=I_{LN}/2I_{S}\;C_{LO}=I_{LO}/I_{S}\;C_{OL/PO}=I_{AL}-2I_{LO}-\;I_{LN}/2I_{S}\;C_{SFA}=S-C_{OL/PO}-C_{LO}-C_{LN}\;UD=I_{AL}/I_{S}$$

where *C_LN_*, *C_LO_*, *C_OL/PO_*, and *C_SFA_* are the proportions of LN, LO, OL+PO, and SFA, respectively. *Is* is the integral of CH_2_α protons signal of FA acyl chains at δ 2.380–2.240, which can be used as an internal reference, and represents the total amount of FA. *I_LN_* is the integral of the bis-allylic protons signal at δ 2.806, *I_LO_* is integral of the bis-allylic protons at δ 2.769, and *I_AL_* is the integral of the allylic protons of OL, PO, and LO at δ 2.125–1.940. For instruments operating in fields higher than 700 MHz, the allylic protons of OL and PO are generally well separated from those of LO; thus, a simpler relationship can be used, namely $C_{OL/PO}=I_{OL/PO}/2I_{S}$, where *I_OL/PO_* is the integral of the allylic protons of OL, PO, and LO at δ 2.029–1.940. The arithmetic coefficients in the relationships are used to normalize for differences in the number of protons associated with each functional group. 

NMR offers a very reproducible and rapid analysis with NMR experimental time that lasts about 6 min, which is an important advantage. The main drawback of NMR compared to other methods such as GC, which is widely used for avocado oil analysis, is that it is not able to discriminate between different SFA or between OL and PO, as already mentioned. Ten representative avocado samples, namely two EV, two R, two U, two PF, and two PW were analyzed. Table 2 summarizes the FA profile data, expressed in relative concentrations, determined in five representative oils used in this study. One sample, labeled as EV, was significantly different from the others in terms of its FA profile, indicating that it is not authentic. For example, its LO content was found to be 55.1%, much higher than the accepted levels for avocado [28]. Its LN was 6.8%, whereas the highest level reported in the literature is 3.9% [10]. For the other samples, there is no specific pattern about the levels of various FA.

### 2.3. Spectral Comparison

**Figure 3:**
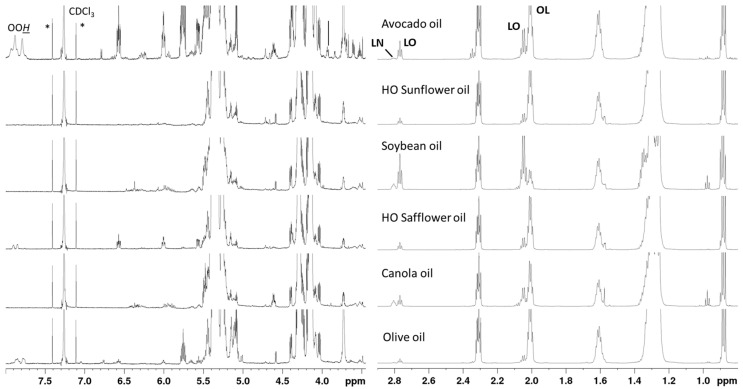
Comparison of the intensity-scaled 700 MHz ^1^H-NMR spectra of avocado oil, HO sunflower oil, soybean oil, HO safflower oil canola oil, and olive oil in CDCl_3_ solutions. *: ^13^C satellites of chloroform.

Figure 3 shows representative intensity-scaled ^1^H-NMR spectra of the oil types used in this study, namely avocado oil, high-oleic (HO) sunflower oil, soybean oil, HO safflower oil, canola oil, and olive oil. The variability in the chemical composition between these oils is also reflected in the spectral characteristics. A visual inspection indicates that there are differences in the FA profile of various oils, for example, in the signals of OL, LO, and LN. Olive oil and avocado oil are known to have many similarities in fatty composition. These similarities are also depicted in the ^1^H-NMR spectra, although more intense signals of the allylic protons of LO and the bis-allylic protons of LN are observed. Variations also exist for several minor compounds, such as oxidation products, especially hydroperoxides with conjugated double bonds. Such compounds have been previously reported for olive oil [29] indicating that the similarities in the FA profile between the two oils results also in a similar chemical profile for oxidation products. Other differences appear in the spectral area where sterols resonate (Figure S2). However, β-sitosterol is the main sterolic peak for all studied oils, while chemical shifts that belong to cyclopropane rings of sterols appear in both olive oil and avocado oil. This analysis indicates that olive oil and avocado oil are expected to form neighboring clusters in the untargeted analysis given their similarities; however, we hypothesize that there could be good separation with proper multivariate statistical analysis.

### 2.4. Untargeted Analysis

**Figure 4:**
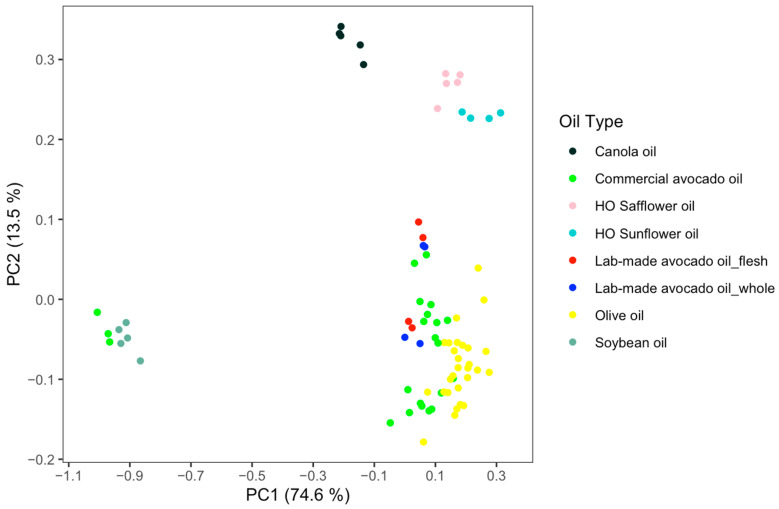
PCA scores-plot of avocado oil, high-oleic (HO) sunflower oil, HO safflower oil, soybean oil, canola oil, and olive oil using 1D- ^1^H-NMR. Three samples labeled as avocado oil samples cluster with soybean oil.

NMR spectroscopy can be combined with chemometrics to determine avocado oil authentication and adulteration with other edible oils. To assess the potential of NMR as a tool for the authentication of avocado oil and to identify its differences from other oils, an untargeted NMR-based metabolomics analysis was applied. The ^1^H-NMR-based untargeted analysis is rapid as less than 6 min are required to record a spectrum with a high signal-to-noise ratio (S/N), even for several minor compounds such as sterols and oxidation products. This feature is important because minor components may be significant markers for clustering and classification. The initial principal component analysis (PCA) plot shown in Figure 4 includes all different oil samples used in this study and helped us to understand compositional differences and similarities between groups. PC1 explains 74.6% and PC2 13.5% of the total variance. NMR was able to achieve a good separation between avocado oil and other types of oil even by using only unsupervised analysis (PCA).

Interestingly, three commercially available avocado oils were clustered with soybean oils indicating a 100% adulteration, although two of them were labeled as extra virgin and one of them was unspecified. These results agree with a previous study where GC was used for the analysis of these samples [13]. The most important biomarkers associated with these samples are LO and LN as found by the bins at δ 2.755–2.850 and δ 2.035–2.065 appearing in the loadings plot. This finding is also in agreement with the FA profile data shown in Table 2 for one of the samples. FA analysis for the other two samples confirmed that they have much lower levels of OL and higher levels of LO and LN. For the unspecified avocado oil sample, OL+PO was found to be 21.2%, LO = 50.6%, and LN = 6.5%, while for the sample labeled as EV, OL+PO = 20.3%, LO = 51.2%, and LN = 8.9%. Avocado oils made by different processing methods, including samples using the whole fruit or only the flesh and samples using different malaxation times and temperatures, were not found to form separate clusters. Further studies focusing on how variables during processing impact avocado oil composition are required to confirm these results. The weakest separation was between avocado oil and olive oil, which have some overlap in the PCA plot; however, the clustering is still considered satisfactory. This result is not surprising due to the compositional similarities between the two products. A PCA using Probabilistic Quotient Normalization (PQN) scaling instead of normalization to total intensity was also tested. However, as shown in Figure S3, clustering was less efficient for some groups; thus, total intensity was selected as the optimum sample scaling approach.

**Figure 5:**
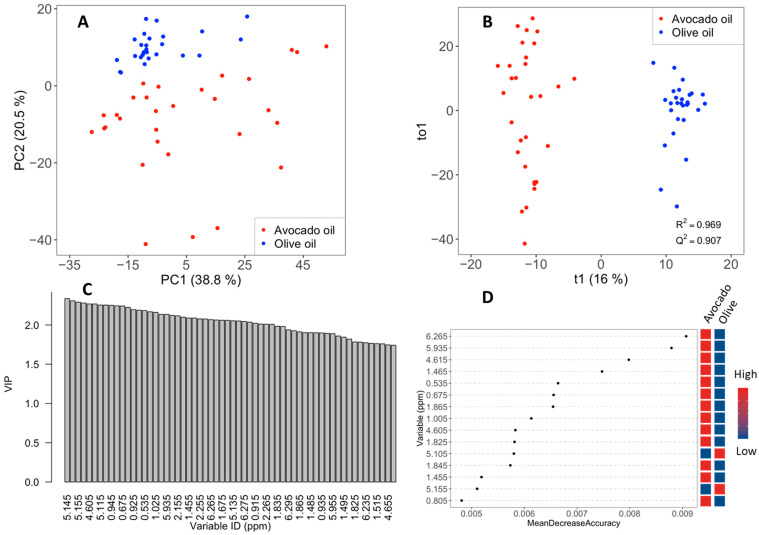
PCA scores-plot of avocado oil and olive oil (**A**). OPLS-DA scores-plot of avocado oil and olive oil (**B**). Variable importance in projection (VIP) plot obtained by OPLS-DA (**C**). Variable importance plot of the fifteen most significant NMR bins obtained by random forest analysis (**D**).

Next, a PCA was conducted only between avocado oil and olive oil samples by removing the three avocado oil samples that were not authentic. Avocado oil and olive oil have a clear separation in this plot, as shown in Figure 5A. The avocado oil samples have more diversity compared to olive oil samples, which appear to form a tighter cluster. This diversity is probably because avocados are processed all year long, so there are more variations in maturations and ripeness of the fruit. Further, there are more ways to process avocados than olives (e.g., flesh vs. whole). PC1 explains 38.9% and PC2 20.5% of the total variance. Interestingly, the two groups were separated along PC2 rather than PC1. Because PCA includes the total variance of a system, this result indicates that other factors, in addition to the type of oil, significantly affect the composition and contribute to the total variance. Loadings that were found to strongly correlate with olive oil were bins at δ 5.09–5.16 and δ 1.68–1.67, which belong to squalene, as well as bins that were assigned to secondary oxidation products such as alkenals (δ 9.63). Bins at δ 2.75–2.83 that belong to bis-allylic protons of LO and LN were associated with avocado oil. In addition, bins at δ 4.05–4.00 that belong to hydrolysis products, namely 1,3-DG, and bins at δ 6.00–6.57, assigned to hydroperoxides, were also associated to avocado oils.

A comparative supervised analysis, namely D, focusing only on the variance arising from the type of oil was conducted. Orthogonal projection to latent structures discriminant analysis (OPLS-DA) scores plot is shown in Figure 5B and classifies the two groups very efficiently with an R^2^ of 0.97, a Q^2^ of 0.91, and a CV-ANOVA *p*-value of 3.2 × 10^−19^, indicating a very significant model. In addition, the R^2^ and Q^2^ values for permutated model were found to be 0.48 and −0.81, respectively, further confirming the high quality of the model. The variable importance in projection (VIP) plot derived from OPLS-DA was used to determine the most important descriptors of the classification. Figure 5C shows the most significant bins in the VIP plot, and Table S1 contains the NMR bins with a VIP value higher than 1. Characteristic examples of variable trend plots showing markers associated with each group are shown in Figure S4. Significant features associated with olive oil include bins that belong to squalene (5.100–5.165). Features associated with avocado oil include bins at δ 4.595–4.655, which are attributed to hydrogen H3 of esterified sterols and δ 1.85, which belong to sterols. The bins at δ 1.015–1.025, which also have large VIP values, have been also assigned to esterified sterols, further supporting this observation. Other bins associated with avocado oil include those of hydroperoxides (6.265, 5.935) and bins at δ 0.535–0.545, which belong to Δ7 sterols, mainly Δ7 avenasterol. It is worth noting that classification with random forest analysis also generated an excellent classification with an out-of-bag error equal to 0.01 and a similar profile for the variable importance as shown in Figure 5D and Table S2.

## 3. Materials and Methods

### 3.1. Samples and Chemicals

Seventy-seven edible oils of six categories, namely avocado (30), canola (5), olive (28), soybean (5), high oleic safflower (5), and high oleic sunflower oils (4) were used in this study. Fourteen avocado oils were obtained from local grocery stores, and eight were purchased online. Details of these samples can be found in another publication [13]; EV, R, and U denote for extra virgin, refined, and unspecified, respectively. In addition to the commercial samples, four samples were produced using only the flesh of avocados (PF), and four were produced using the whole avocado fruits (PW) with a laboratory-scale oil extraction system (Abencor analyzer, MC2 Ingenieria y Sistemas S.L., Seville, Spain) in the Wang Lab. Chloroform-d1 (CDCl_3_) was purchased from Cambridge Isotope Laboratories (Tewksbury, MA, USA).

### 3.2. Sample Preparation for NMR Experiments

Oil samples were randomized and prepared under an identical protocol. Briefly, 20 mg of oil were placed into a 4 mL vial. The sample was dissolved in 600 μL of CDCl_3_ containing 0.01% of tetramethylsilane (TMS), and the solution was transferred into a 5 mm NMR tube. All NMR samples were stored under the same storage conditions and were analyzed within 24 h after preparation.

### 3.3. NMR Experiments

^1^H- and ^13^C-NMR experiments were conducted on a Bruker Avance III spectrometer (Bruker, Ettlingen, Germany) operating at 700.13 MHz for ^1^H nucleus, equipped with a TXO helium-cooled 5-mm probe. All experiments were performed at 25 ± 0.1 °C, and the spectra were processed by the Topspin software package provided by Bruker Biospin. ^1^H-NMR experiments were conducted using the following acquisition parameters: 64 scans 64K data points, acquisition time 3.12 s, 90° pulse angle (10.8 μs), relaxation delay 1 s, and spectral width 15 ppm. A polynomial fourth-order function was applied for base-line correction in order to achieve accurate quantitative measurements upon integration of signals of interest. The spectra were acquired without spinning the NMR tube in order to avoid artifacts, such as spinning side bands of the first or higher order. Chemical shifts are reported in ppm from TMS (δ = 0).

The ^1^H-^1^H Total Correlation Homonuclear Spectroscopy (TOCSY) experiments were run in the phase-sensitive mode with States-TPPI mode, using the DISPI2 pulse sequence for the spin lock with 2K and 512 data points in F1 and F2, respectively, 32 scans, 64 dummy scans, 90° pulse angle, spin-lock time of 80 ms, relaxation delay 1.5 s, and spectral width 12 in both dimensions. The NOAH-BSC experiment was performed using 2K and 768 data points in F1 and F2, respectively, and were processed using the splitx AU program from Bruker user library to separate the data.

### 3.4. Multivariate Data Analysis

For ^1^H-NMR untargeted analysis, the spectral regions δ 10.00−0.50 were integrated into regions (bins) with equal width of 0.01 ppm using the AMIX software package (V3.9, Bruker topspin, Billerica, MA, USA). To correct for potential concentration differences among samples and/or NMR pulse variations, the bucketed regions were normalized to the total sum of the spectral integrals before applying statistics. Multivariate statistical analysis (MVSA), namely PCA, OPLS-DA, and Random Forest was carried out in R environment (RStudio, Version 4.0.2, Boston, MA, USA) using “prcomp”, “ropls”, “random forest”, ggplot” and “tidyverse” packages.

## 4. Conclusions

This is the first application of NMR spectroscopy for the characterization and assessment of avocado oil, in which high-resolution ^1^H-NMR was demonstrated to be an efficient and rapid tool. The relative amounts of various FA can be easily determined, although discrimination between OL and PO is not feasible. Combined with chemometrics, ^1^H-NMR allowed for the successful clustering and classification of avocado oil and other edible oils. Interestingly, NMR detected the adulteration in three products labeled as avocado oils, which were clustering together with soybean oil, confirming previous findings [13]. This indicates purity issues in commercially available avocado oils. Untargeted analysis showed that the oil with the highest similarity to avocado oil was olive oil. However, there are remarkable differences among them, especially for squalene, which is found mostly in olive oil, and sterolic compounds, which are found more in avocado oil.

## Figures and Tables

**Table 1:** ^1^H-NMR chemical shifts of the main avocado oil constituents in CDCl_3_ solution.

Signal	δ (ppm)	Functional Group	Component
1	0.880	CH_3_CH_2_ (t), *J* = 7.1 Hz	SFA, OL
2	0.883	CH_3_CH_2_ (t), *J* = 7.2 Hz	LO
3	0.976	CH_3_CH_2_ (t), *J* = 7.5 Hz	LN
4	1.145–1.450	-(CH_2_)_n_- (envelope)	All acyl chains
5	1.612	-CH_2_(β), m	All acyl chains
6	2.009	CH_2_CH=CH (m)	OL
7	2.048	CH_2_CH=CH (m)	LO, LN
8	2.308	-CH_2_(α) (m)	TG, All acyl chains
9	2.347	-CH_2_α (t), *J* = 7.6 Hz	DG, 1-MG, FFA All acyl chains
10	2.769	CH=CHCH_2_CH=CH (m)	LO
11	2.805	CH=CHCH_2_CH=CH (br)	LN
12	3.602	3’a-CH_2_OCO (dd)	1-MG
13	3.690	3’a-CH_2_OCO (dd)	1-MG
14	3.730	3’a, 3’b-CH_2_OCO	1,2-DG
15	3.839	1’a,’b, 3’a,’b HOCH_2_-CH(OR)-CH_2_OH (m)	2-MG
16	3.935	2’-CHOH	1-MG
17	4.080	2’-CHOH (br)	1,3 DG
18	4.144	CH_2_OCO (dd) *J* = 11.96 Hz *J* = 4.35 Hz	TG
19	4.185	1’b, 3’b-CH_2_OCO (dd) *J* = 11.35 Hz *J* = 4.35 Hz	1,3 DG
20	4.295	CH_2_OCO (dd) *J* = 11.96 Hz *J* = 4.35 Hz	TG
21	5.083	2’-CHOCO (m)	1,2-DG
22	5.264	2’-CHOCO (m)	TG
23	5.300–5.400	CH=CH (m)	All acyl chains

**Table 2:** FA composition (%) of ten commercially available and lab-made avocado oil samples as determined by ^1^H-NMR spectroscopy.

Component	1	2	3	4	5	6	7	8	9	10
OL/PO	60.7	21.5	72.0	70.6	70.2	67.7	67.9	66.2	70.6	64.6
LO	18.9	55.1	15.7	12.8	10.6	16.8	16.3	16.8	15.3	18.7
LN	0.9	6.8	0.5	0.6	0.6	0.4	1.1	0.9	1.0	1.1
SFA	19.5	16.6	11.8	16.0	18.6	15.1	12.6	16.1	13.1	15.6
UD	78.05	76.5	85.3	81.3	80.9	82.7	84.1	80.5	83.9	81.4

1, EV; 2, EV; 3, R; 4, R, 5, U; 6, U; 7, PF; 8, PF; 9, PW; 10, PW. EV, R, U, PF, and PW denote extra virgin, refined, unspecified, made by the flesh of avocado, and made by the whole fruit, respectively.

## Data Availability

The data presented in this study are available in article and supplementary material.

## Supplementary Materials

The following are available online. Figure S1. HSQC-DEPT (A) and HMBC (B) spectra of avocado oil in the peroxide area. Figure S2. Title: Comparison of the intensity-scaled 700 MHz ^1^H-NMR spectra of avocado oil, HO sunflower oil, soybean oil, HO safflower oil canola oil, and olive oil in the spectral area where sterols resonate. Figure S3. Title: PCA scores-plot avocado oil, high-oleic (HO) sunflower oil, soybean oil, HO safflower oil canola oil using Probabilistic Quotient Normalization (PQN) scaling. Figure S4. Title: Variable trend plots obtained by OPLS-DA model for squalene, esterified sterols, hydroperoxides and β-sitosterol. Table S1: title NMR bins with a VIP score > 1, obtained by OPLS-DA. Table 2: confusion matrix obtained by random forest analysis.

## Sample Availability

Samples of the compounds are not available from the authors.

## References

[1] Werman M.J., Neeman I. (1987). Avocado oil production and chemical characteristics. J. Am. Oil Chem. Soc..

[2] Terés S., Barceló-Coblijn G., Benet M., Álvarez R., Bressani R., Halver J.E., Escribá P.V. (2008). Oleic acid content is responsible for the reduction in blood pressure induced by olive oil. Proc. Natl. Acad. Sci. USA.

[3] Altendorf S. (2019). Major Tropical Fruits Market Review 2018.

[4] Flores M., Saravia C., Vergara C.E., Avila F., Valdés H., Ortiz-Viedma J. (2019). Avocado Oil: Characteristics, Properties, and Applications. Molecules.

[5] Moreno A.O., Dorantes L., Galíndez J., Guzmán R.I. (2003). Effect of Different Extraction Methods on Fatty Acids, Volatile Compounds, and Physical and Chemical Properties of Avocado (Persea americana Mill.) Oil. J. Agric. Food Chem..

[6] Corzzini S.C.S., Barros H.D.F.Q., Grimaldi R., Cabral F.A. (2017). Extraction of edible avocado oil using supercritical CO_2_ and a CO_2_/ethanol mixture as solvents. J. Food Eng..

[7] Costagli G., Betti M. (2015). Avocado oil extraction processes: Method for cold-pressed high-quality edible oil production versus traditional production. J. Agric. Eng..

[8] Martínez-Padilla L.P., Franke L., Xu X.-Q., Juliano P. (2018). Improved extraction of avocado oil by application of sono-physical processes. Ultrason. Sonochem..

[9] Fernandes G.D., Gómez-Coca R.B., Pérez-Camino M.C., Moreda W., Barrera-Arellano D. (2018). Chemical characterization of commercial and single-variety avocado oils. Grasas Aceites.

[10] Tan C.X., Tan S.S., Tan S.T. (2017). Influence of Geographical Origins on the Physicochemical Properties of Hass Avocado Oil. J. Am. Oil Chem. Soc..

[11] Yanty N.A.M., Marikkar J.M.N., Long K. (2011). Effect of Varietal Differences on Composition and Thermal Characteristics of Avocado Oil. J. Am. Oil Chem. Soc..

[12] Donetti M., Terry L.A. (2014). Biochemical markers defining growing area and ripening stage of imported avocado fruit cv. Hass. J. Food Compos. Anal..

[13] Green H.S., Wang S.C. (2020). First report on quality and purity evaluations of avocado oil sold in the US. Food Control.

[14] Sacchi R., Medina I., Aubourg S.P., Addeo F., Paolillo L. (1993). Proton nuclear magnetic resonance rapid and structure-specific determination ofω-3 polyunsaturated fatty acids in fish lipids. J. Am. Oil Chem. Soc..

[15] Igarashi T., Aursand M., Hirata Y., Gribbestad I.S., Wada S., Nonaka M. (2000). Nondestructive quantitative determination of docosahexaenoic acid and n−3 fatty acids in fish oils by high-resolution 1H nuclear magnetic resonance spectroscopy. J. Am. Oil Chem. Soc..

[16] Tang F., Vasas M., Hatzakis E., Spyros A. (2019). Magnetic resonance applications in food analysis. Annu. Rep. NMR Spectrosc..

[17] Hatzakis E. (2019). Nuclear Magnetic Resonance (NMR) Spectroscopy in Food Science: A Comprehensive Review. Compr. Rev. Food Sci. Food Saf..

[18] Vigli G., Philippidis A., Spyros A., Dais P. (2003). Classification of Edible Oils by Employing ^31^P and ^1^H NMR Spectroscopy in Combination with Multivariate Statistical Analysis. A Proposal for the Detection of Seed Oil Adulteration in Virgin Olive Oils. J. Agric. Food Chem..

[19] Kupče Ē., Claridge T.D.W. (2019). New NOAH Modules for Structure Elucidation at Natural Isotopic Abundance. J. Magn. Reson..

[20] Tang F., Hatzakis E. (2020). NMR-Based Analysis of Pomegranate Juice Using Untargeted Metabolomics Coupled with Nested and Quantitative Approaches. Anal. Chem..

[21] Ahmed R., Varras P.C., Siskos M.G., Siddiqui H., Choudhary M.I., Gerothanassis I.P. (2020). NMR and Computational Studies as Analytical and High-Resolution Structural Tool for Complex Hydroperoxides and Diastereomeric Endo-Hydroperoxides of Fatty Acids in Solution-Exemplified by Methyl Linolenate. Molecules.

[22] Prescha A., Grajzer M., Dedyk M., Grajeta H. (2014). The Antioxidant Activity and Oxidative Stability of Cold-Pressed Oils. J. Am. Oil Chem. Soc..

[23] Salvo A., Rotondo A., Torre G.L.L., Cicero N., Dugo G. (2017). Determination of 1,2/1,3-diglycerides in Sicilian extra-virgin olive oils by ^1^H-NMR over a one-year storage period. Nat. Prod. Res..

[24] Guillén M.D., Ruiz A. (2003). Rapid simultaneous determination by proton NMR of unsaturation and composition of acyl groups in vegetable oils. Eur. J. Lipid Sci. Technol..

[25] Castejón D., Fricke P., Cambero M.I., Herrera A. (2016). Automatic ^1^H-NMR Screening of Fatty Acid Composition in Edible Oils. Nutrients.

[26] Williamson K., Hatzakis E. (2019). NMR analysis of roasted coffee lipids and development of a spent ground coffee application for the production of bioplastic precursors. Food Res. Int..

[27] Dais P., Misiak M., Hatzakis E. (2015). Analysis of marine dietary supplements using NMR spectroscopy. Anal. Methods.

[28] Manaf Y.N., Rahardjo A.P., Yusof Y.A., Desa M.N., Nusantoro B.P. (2018). Lipid characteristics and tocopherol content of the oils of native avocado cultivars grown in Indonesia. Int. J. Food Prop..

[29] Ruiz-Aracama A., Goicoechea E., Guillén M.D. (2017). Direct study of minor extra-virgin olive oil components without any sample modification. 1H NMR multisupression experiment: A powerful tool. Food Chem..

