# Supplementary material for: Analysis and Authentication of Avocado Oil Using High Resolution NMR Spectroscopy
Source: Molecules. 2021 Jan 9;26(2):310. doi: 10.3390/molecules26020310 (PMC7828049; doi:10.3390/molecules26020310)
Supplement: Supplementary file 1 [file molecules-26-00310-s001.pdf]

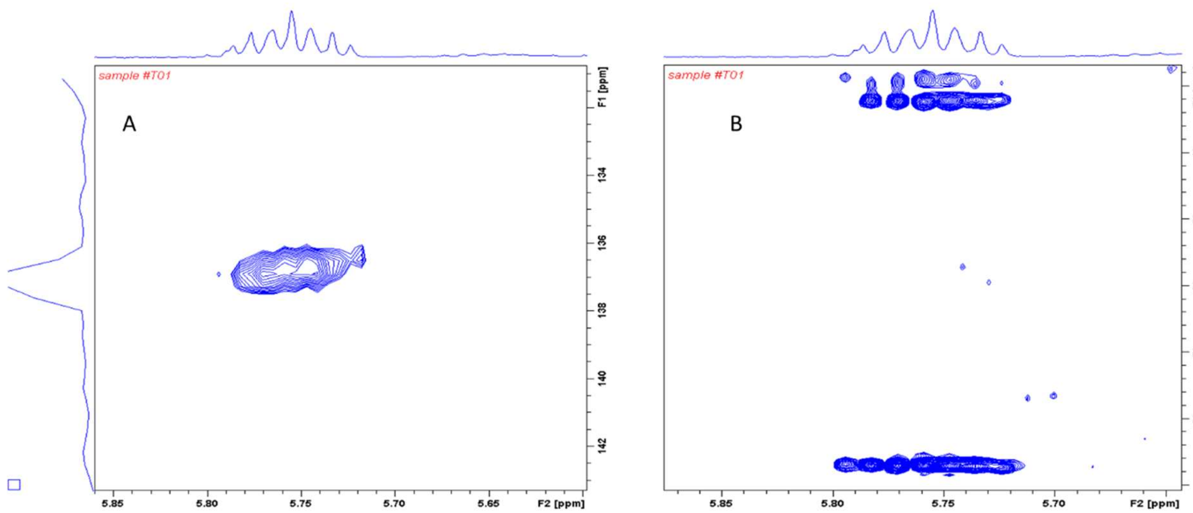

Figure S1. HSQC-DEPT (A) and HMBC (B) spectra of avocado oil in the peroxide area.

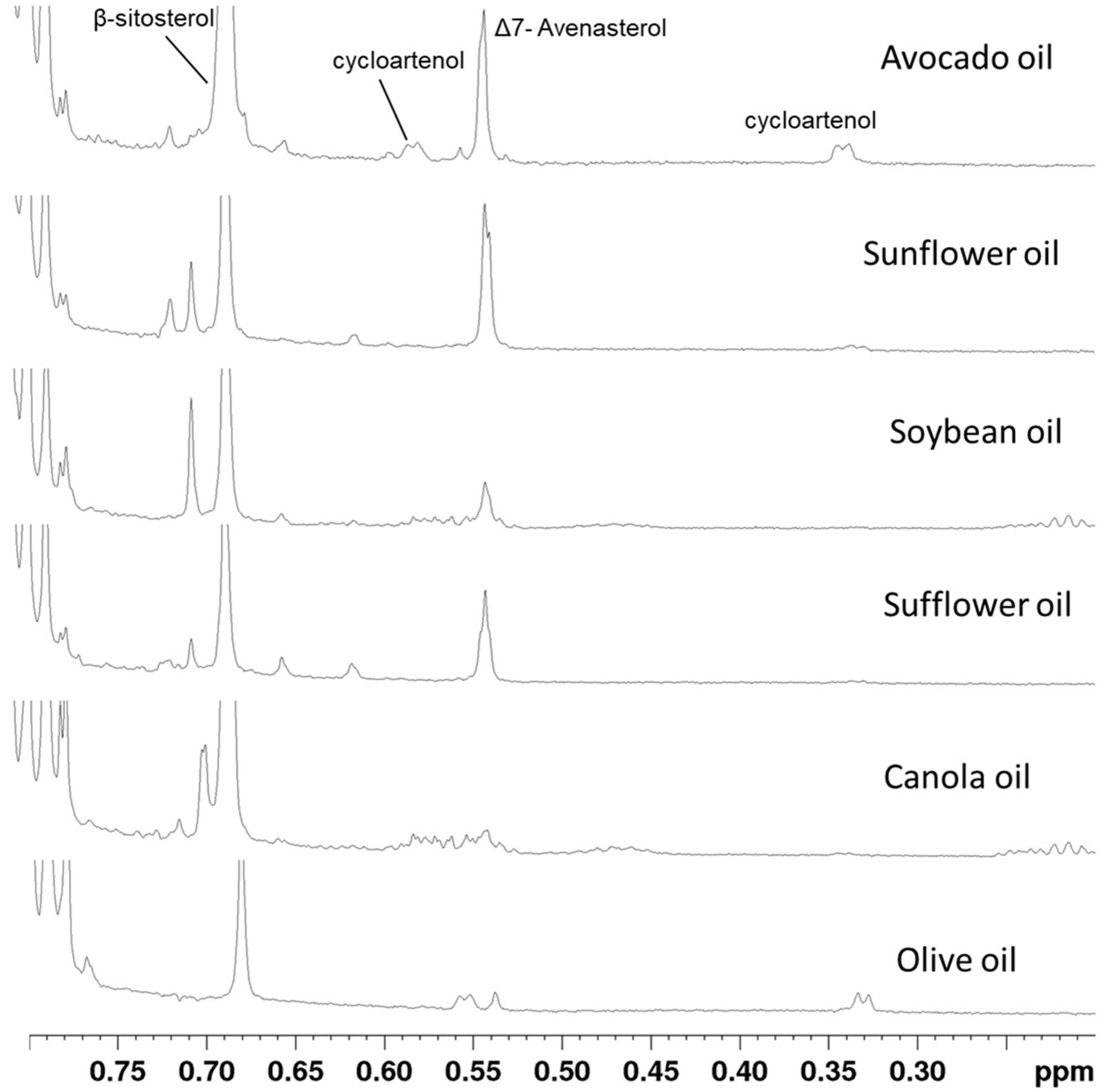

Figure S2. Comparison of the intensity-scaled 700 MHz 1H-NMR spectra of avocado oil, HO sunflower oil, soybean oil, HO safflower oil canola oil, and olive oil in the spectral area where sterols resonate.

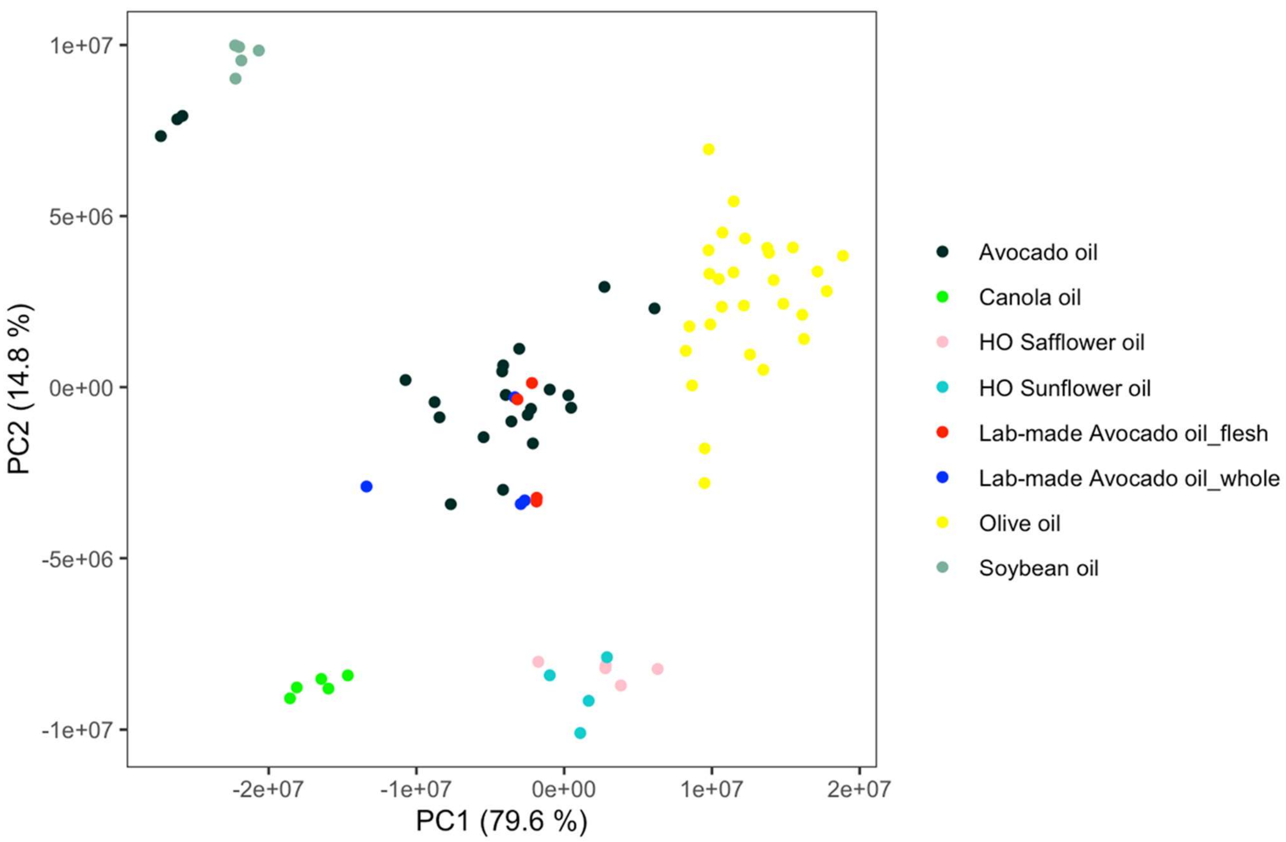

Figure S3. PCA scores-plot avocado oil, high oleic (HO) sunflower oil, soybean oil, HO safflower oil canola oil using Probabilistic Quotient Normalization (PQN) scaling.

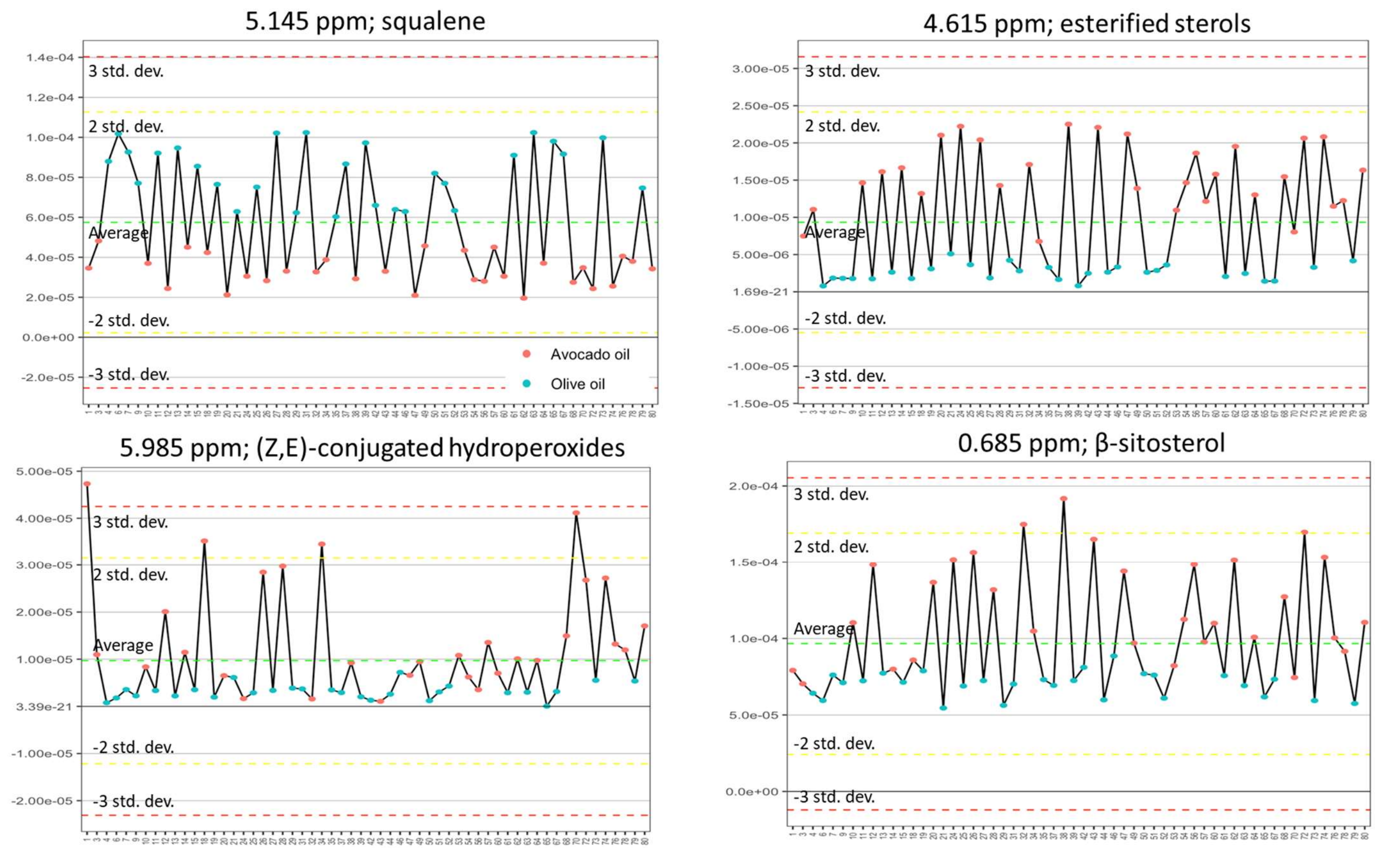

Figure S4. Variable trend plots obtained by OPLS-DA model for squalene, esterified sterols, hydroperoxides and β-sitosterol.

Table S1. NMR bins with a VIP score > 1, obtained by OPLS-DA.

**Bin (δ)**	**VIP**
5.145	2.3339093
1.015	2.30589463
5.155	2.28848021
5.105	2.27758019
4.605	2.26559212
4.615	2.26525195
5.115	2.25116449
4.625	2.25083701
0.945	2.24797577
6.285	2.24107534
0.675	2.23895845
0.805	2.221259
0.925	2.19507396

4.635	2.18774009
0.535	2.18363501
4.725	2.1674145
1.025	2.16032258
1.315	2.13523456
5.935	2.13459681
0.815	2.12151031
2.155	2.11647646
5.125	2.0978578
1.455	2.08838039
0.845	2.08820981
2.255	2.07717254
6.245	2.07571552
6.265	2.06751044
0.835	2.06302307
1.675	2.05984059
1.845	2.0588028
5.135	2.05320361
1.685	2.05038245
6.275	2.04414045
2.165	2.03481979
0.915	2.02171006
1.005	2.01107338
2.265	2.00977926
1.465	2.00791765
1.835	1.98279124
1.855	1.98028584
6.295	1.9372572
6.305	1.9264635
1.865	1.91226715
1.445	1.90151882
1.485	1.90148731
1.475	1.90094586
0.935	1.89830929
0.955	1.89316699
5.955	1.88781785
0.685	1.85867932
1.495	1.84409409
3.585	1.82064745
1.825	1.78381596
4.645	1.78041507

6.235	1.773102
5.945	1.76456455
1.515	1.76133254
2.245	1.75908669
4.655	1.74629924
9.655	1.7415056
1.975	1.73417757
1.525	1.73180306
2.145	1.71869287
1.245	1.71745452
0.825	1.71675519
4.705	1.71226343
9.635	1.70672533
5.095	1.68782117
3.525	1.68613878
3.535	1.68344939
5.925	1.68281537
1.505	1.68045279
3.845	1.67467008
1.665	1.65959695
0.995	1.65926826
1.435	1.65143878
1.625	1.6465921
1.985	1.64583161
2.175	1.64447691
1.385	1.61266419
3.675	1.61127141
2.335	1.61072899
6.755	1.6103804
1.235	1.60483608
6.765	1.60454935
3.515	1.59945565
4.565	1.59903535
1.115	1.59510155
2.235	1.58785685
1.635	1.57399281
2.375	1.57137535
3.835	1.57136823
7.045	1.56992936
3.595	1.56862398
3.915	1.55605836

9.235	1.54220562
1.815	1.53674806
3.605	1.52897524
2.445	1.51948957
3.665	1.50605802
1.105	1.50010337
2.455	1.49940499
2.365	1.49727238
5.985	1.48723274
2.185	1.48239951
1.655	1.47016386
4.595	1.46219057
2.355	1.45907677
7.055	1.45906232
3.615	1.45807075
3.705	1.45601811
4.245	1.45081123
2.385	1.45058699
2.345	1.45009512
5.995	1.448973
4.385	1.44545156
4.695	1.44542295
2.305	1.43749666
1.645	1.43676394
3.545	1.42823091
2.195	1.42520969
3.505	1.42291923
1.075	1.42212026
5.575	1.41910286
6.025	1.41472214
4.375	1.40897937
1.085	1.40784188
9.215	1.40697132
2.275	1.40278265
0.865	1.40272451
2.425	1.40196519
5.555	1.40080824
4.365	1.39958403
6.555	1.39923092
6.005	1.39761413
7.915	1.39621778

6.015	1.39524269
2.225	1.39466289
6.545	1.39452197
6.255	1.39433312
4.225	1.39368704
2.435	1.3886441
7.905	1.3881613
1.265	1.38782418
6.045	1.38686612
6.575	1.38625448
4.095	1.3836402
5.585	1.38056124
4.415	1.38051101
9.515	1.37769254
2.465	1.37637253
4.735	1.37565062
4.395	1.37336293
4.235	1.36771889
6.565	1.36726391
4.085	1.36305118
1.395	1.36021401
4.925	1.35928921
6.035	1.35633643
4.195	1.35055544
6.055	1.35052461
2.475	1.35011009
5.565	1.34922912
9.645	1.34805016
1.095	1.34559175
4.325	1.34338459
3.945	1.34203715
6.225	1.34126692
4.425	1.3377073
1.375	1.33588009
4.185	1.33490544
1.875	1.32829643
3.935	1.32747721
6.595	1.32724818
3.875	1.32593399
3.695	1.32540044
4.355	1.32324024

3.735	1.32041198
1.955	1.32031668
3.925	1.31594375
6.585	1.31330098
5.475	1.3130591
3.575	1.31061911
5.485	1.31056443
5.545	1.30729365
5.975	1.30391663
3.975	1.30308876
5.285	1.30259868
4.175	1.30256373
1.945	1.3019471
5.465	1.30055468
6.315	1.29696758
5.175	1.29661092
4.405	1.28840798
5.495	1.28650909
3.725	1.28620283
4.315	1.28482902
4.755	1.2825481
7.895	1.27683764
2.205	1.27589718
5.505	1.26748041
2.115	1.26627955
4.075	1.26625801
1.605	1.26169944
2.135	1.26061689
5.915	1.26017669
2.325	1.25939947
4.065	1.25611909
4.875	1.25448742
1.255	1.252741
3.685	1.24774251
0.875	1.2477052
7.925	1.2400917
5.515	1.23637218
5.965	1.23322712
1.425	1.22245781
1.225	1.22218696
4.555	1.2182103

4.685	1.21775694
4.935	1.21400999
2.415	1.21363244
7.965	1.21212651
3.885	1.21118007
3.455	1.20747704
9.685	1.20719127
5.335	1.20550707
2.485	1.2011275
3.825	1.19991668
5.075	1.19762755
2.125	1.18874315
4.865	1.18767809
0.795	1.18162361
1.595	1.17765788
1.405	1.17637016
4.745	1.17612012
4.215	1.17479686
5.425	1.17472515
5.165	1.17251605
0.895	1.17190755
4.055	1.17111422
1.135	1.16850324
7.865	1.16549964
1.345	1.16452029
9.545	1.16323826
1.055	1.16059282
7.955	1.15913917
6.065	1.15727999
5.645	1.15465305
5.055	1.15343682
2.295	1.15243532
1.995	1.15241271
2.105	1.1501143
7.975	1.14817568
3.565	1.14689181
2.215	1.14453339
5.065	1.14055831
7.985	1.13652496
3.985	1.13115925
5.455	1.13102634

3.995	1.13051387
0.905	1.12695947
2.695	1.12067219
1.365	1.11799367
4.915	1.11760542
7.875	1.11208353
3.445	1.11190012
2.685	1.11086575
1.705	1.10787029
2.665	1.107357
3.285	1.10407894
4.045	1.10271596
7.855	1.10136915
3.465	1.10025621
2.785	1.09978226
4.675	1.09885818
1.355	1.09689713
2.925	1.09387257
4.205	1.09341546
5.435	1.09158465
7.595	1.0871188
2.035	1.08621455
2.775	1.0847772
4.165	1.08471904
2.675	1.08455449
4.105	1.08420271
3.855	1.07870939
4.975	1.07866051
2.905	1.07568424
1.555	1.07358656
9.765	1.07247014
2.935	1.0718498
1.065	1.06709359
3.865	1.06457172
3.555	1.0636735
1.295	1.063026
3.785	1.06142817
1.305	1.05731376
4.335	1.05566856
2.065	1.05472738
2.765	1.05307644

2.865	1.05192713
3.795	1.05112954
5.355	1.04843541
1.275	1.04750058
2.855	1.04660853
2.045	1.04486894
3.655	1.04462357
2.655	1.04204345
3.745	1.04043158
6.075	1.03932445
4.825	1.03864588
2.945	1.03856517
4.835	1.03716162
2.095	1.03637231
1.415	1.03587812
2.005	1.03492708
4.255	1.03374455
2.055	1.03346095
5.375	1.0286436
5.415	1.02768041
5.525	1.02432214
7.885	1.01702939
2.755	1.0154229
4.815	1.01415979
7.945	1.01259475
2.645	1.01207186
7.035	1.00762596
2.955	1.00704192
7.845	1.00375227
2.405	1.00324951
1.725	1.00207804
4.795	0.99776349
0.555	0.99674913

Table S2. Confusion matrix obtained by random forest analysis.

	**Avocado**	**Olive**	**class.error**
Avocado	30	0	0.0
Olive	0	28	0.0
